# Exercise and Lifestyle Modification in the Management of Diabetic Patients in the Al-Madinah Region, Saudi Arabia

**DOI:** 10.7759/cureus.89139

**Published:** 2025-07-31

**Authors:** Osman Suliman, Ali A Fadhel, Ibrahim S Aljohani, Turki A AAlsenan, Abdullah K Alharbi, Khalid E Howladr, Mohammed M Albensari, Khalid H Almutairi, Khaled M AboZena, Magda A Eldomiaty

**Affiliations:** 1 Clinical Sciences, Al-Rayan National College of Medicine, Al-Rayan National Colleges, Al-Madinah, SAU; 2 Medicine and Surgery, Al-Rayan National College of Medicine, Al-Madinah, SAU; 3 Anatomy, Embryology, and Histology, Al-Rayan National College of Medicine, Al-Madinah, SAU

**Keywords:** al-madinah, exercise, lifestyle modification, nutrition, saudi arabia, type 2 diabetes

## Abstract

Background: Diabetes is a public health problem in Saudi Arabia, and the disease outcomes are highly affected by lifestyle factors such as diet and physical activity.

Objective: The current study aimed to examine the role of culturally sensitive lifestyle modifications in controlling type 2 diabetes in Al-Madinah.

Methods: We conducted a retrospective cross-sectional analysis of electronic health records among 381 adults with type 2 diabetes at two large hospitals in Al-Madinah. Data on demographic characteristics, dietary habits, and physical activity were obtained. Logistic regression was employed to analyze correlations between lifestyle and health status (e.g., own perceived energy level) and reporting odds ratios (ORs).

Results: Of the patients, 54.9% exercised regularly, and walking was the most common exercise. Furthermore, 65.6% of the patients reported dietary changes to manage diabetes; the most frequently reported changes were a reduction in the consumption of sugars/processed foods (49%) and carbohydrates (38%). Patients who exercise had more than 2.89 times (p < 0.001) the odds of reporting satisfaction with energy levels. In general, uptake of lifestyle change was good and appeared beneficial.

Conclusion: These findings suggest that Saudi patients readily adopt culturally sensitive exercise and dietary modifications and that such behaviors are associated with well-being. Tailoring interventions to cultural norms, e.g., promoting walking and so-called "traditional" healthy foods, may improve diabetes control. Overall, our results support the inclusion of culturally specific lifestyle advice in diabetes management in Saudi Arabia. This has important implications for public health policy, and there is a demand for culturally appropriate interventions to improve outcomes in this high-risk population.

## Introduction

Diabetes has become a major public health issue in Saudi Arabia, with rising prevalence closely linked to lifestyle trends such as inappropriate dietary habits, physical inactivity, and increasing obesity. These factors are particularly concerning in Saudi society, where fast-food consumption and cultural dietary practices contribute significantly to poor health outcomes [1]. This study emphasizes the urgent need for culturally appropriate diabetes management strategies, particularly those focused on improving nutrition and physical activity, which have been shown to enhance patient outcomes and glycemic control [2].

Despite global evidence supporting lifestyle interventions for diabetes, there remains a significant knowledge gap within the Saudi context. The lack of culturally tailored treatment plans, along with limited understanding of patient adherence, poses a barrier to effective diabetes control. This study aims to address this gap by evaluating the feasibility and impact of culturally specific interventions, including nutrition and physical activity programs, on diabetes care and patient compliance in the Al-Madinah Region [3].

Both lifestyle and exercise changes are strongly supported by evidence as effective strategies for improving glucose control and reducing complications [4]. However, most research in the Al-Madinah Region has not adequately considered the socioeconomic, cultural, and environmental factors influencing the adoption of such behaviors. Cultural traditions, dietary norms, and social expectations around physical activity often hinder the implementation of sustainable lifestyle changes [5].

Food plays a significant role in Saudi social and cultural gatherings, often involving high-calorie traditional dishes and limited intake of fruits and vegetables. This dietary pattern contributes to obesity and type 2 diabetes risk [6]. Although awareness of healthy eating is increasing, adherence remains low due to strong cultural ties to traditional cuisine [7].

Cultural norms also present obstacles to regular physical activity, especially among women, who face social constraints and limited access to gender-segregated fitness facilities. Although recent developments such as the establishment of women-only gyms and government initiatives have improved access, barriers to widespread exercise adoption remain [8].

This study aimed to explore practical, culturally aligned strategies for promoting physical activity among diabetic patients in Al-Madinah. These included mobile health (mHealth) apps offering tailored fitness plans, community-based programs such as walking clubs in air-conditioned malls, and mass education campaigns led by media, religious leaders, and community influencers. Such approaches aim to address both cultural and logistical challenges to physical activity [9].

Western treatment models often fail to align with Saudi cultural contexts. This research sought to bridge that gap by integrating traditional dietary practices and family-centered care with modern, evidence-based interventions. For instance, dietary therapies were adapted to include familiar Saudi foods, making them more acceptable and sustainable. The study also examined the role of family and social networks in reinforcing long-term behavioral change [10].

The overarching goal was to develop a culturally sensitive, evidence-based model of diabetes care in Saudi Arabia. This model is designed to enhance patient education, support self-care, and foster lasting adherence to lifestyle modifications. Tools such as electronic monitoring, community support, and culturally relevant diet and exercise plans were central to this framework [11].

By aligning global best practices with local cultural knowledge, this research aims to improve patient outcomes, reduce long-term complications such as renal failure and cardiovascular disease, and enhance the quality of life for diabetics in Al-Madinah [12]. The study aims to evaluate the impact of lifestyle modifications, including exercise and diet, on the management of type 2 diabetes among patients in the Al-Madinah Region.

## Materials and methods

Study design

This retrospective cross-sectional study was conducted among patients with type 2 diabetes who received care at King Fahad Hospital and Al-Madinah General Hospital in the Al-Madinah Region, Saudi Arabia. Demographic characteristics, lifestyle behaviors, and diabetes control methods, including dietary patterns, physical activity levels, and glycemic management practices, were recorded using an electronic data collection sheet. The study investigated the frequency of lifestyle modifications among diabetic patients and explored their association with self-reported indicators of well-being and diabetes self-management, rather than clinical outcomes or complications.

Setting and time period

The study was conducted in the Al-Madinah Region, Saudi Arabia, from March to June 2025.

Inclusion and exclusion criteria and sampling technique

Adults aged 18-65 years with a confirmed diagnosis of type 2 diabetes, as verified by medical records, were included in the study. All participants were required to provide informed consent, not be pregnant, and be capable of completing all aspects of the data collection process, including self-reported lifestyle information and follow-up assessments. Exclusion criteria included having type 1 diabetes or other types of diabetes, severe comorbidities such as advanced cardiovascular or renal disease, current pregnancy or plans for pregnancy, participation in other clinical trials, or cognitive impairment that hindered consent or participation.

Study population

The study population consisted of type 2 diabetes patients attending outpatient departments at King Fahad Hospital and Al-Madinah General Hospital. A control group was also included, comprising patients receiving routine diabetes care without culturally adapted interventions. Participants in both groups were matched based on age, sex, and disease duration to ensure comparability.

Data collection methods

Data were collected using structured questionnaires and electronic data collection forms. The demographic information section included age, gender, ethnicity, and place of residence (urban or rural). The clinical information section documented the diagnosis and duration of type 2 diabetes, family history, complications (e.g., neuropathy and retinopathy), and treatment modalities (e.g., oral medications and insulin therapy). The lifestyle and dietary factors section captured data on body mass index (BMI), dietary intake (fiber, processed food, and sugary beverages), and physical activity levels. The socioeconomic status section included income, occupation, and educational attainment. The healthcare access section recorded the frequency of healthcare visits, time to receive care, and healthcare facility location. The additional information section included smoking and alcohol consumption, where applicable.

Data analysis

Data were analyzed using the Statistical Package for the Social Sciences (SPSS) version 22.0 (IBM Corp., Armonk, NY). Descriptive statistics were used to summarize demographic data. Chi-square tests and correlation analyses were performed to explore associations between variables. A p-value of less than 0.05 was considered statistically significant.

Ethical considerations

The study protocol was approved by the Research Ethics Committee of Al-Rayan National Colleges (approval number: HA-03-M-122-123). Informed consent was obtained from all participants. Data confidentiality and participant privacy were strictly maintained. All data were anonymized and stored on a password-protected laptop accessible only to the research team. Data will be retained securely for three years following the conclusion of the study.

## Results

The mean duration of diabetes was 5.95 ± 4.43 years. The largest age groups were 41-50 years (90, 23.6%), followed by 51-60 years (85, 22.3%), while those under 20 years constituted 29 (7.6%) of the sample. There were 205 (53.8%) male participants and 176 (46.2%) female participants. Regarding occupation, the majority were unemployed (144, 37.8%) or employed full-time (97, 25.5%). For the duration of diabetes, 96 (25.2%) participants had diabetes for less than one year, 87 (22.8%) for 1-5 years, 105 (27.6%) for 6-10 years, and 93 (24.4%) for more than 10 years, as shown in Table 1.

**Table 1:** Distribution of study participants according to their demographic and clinical characteristics (N = 381)

Characteristics	Frequency	Percentage
Age in years		
<20	29	7.6
21-30	48	12.6
31-40	72	18.9
41-50	90	23.6
51-60	85	22.3
>60	57	15
Gender		
Male	205	53.8
Female	176	46.2
Occupation		
Student	47	12.3
Employee (part time)	42	11
Employee (full time)	97	25.5
Employer	51	13.4
Unemployed	144	37.8
Duration of diabetes		
<1 year	96	25.2
1-5 years	87	22.8
6-10 years	105	27.6
>10 years	93	24.4

**Figure 1:**
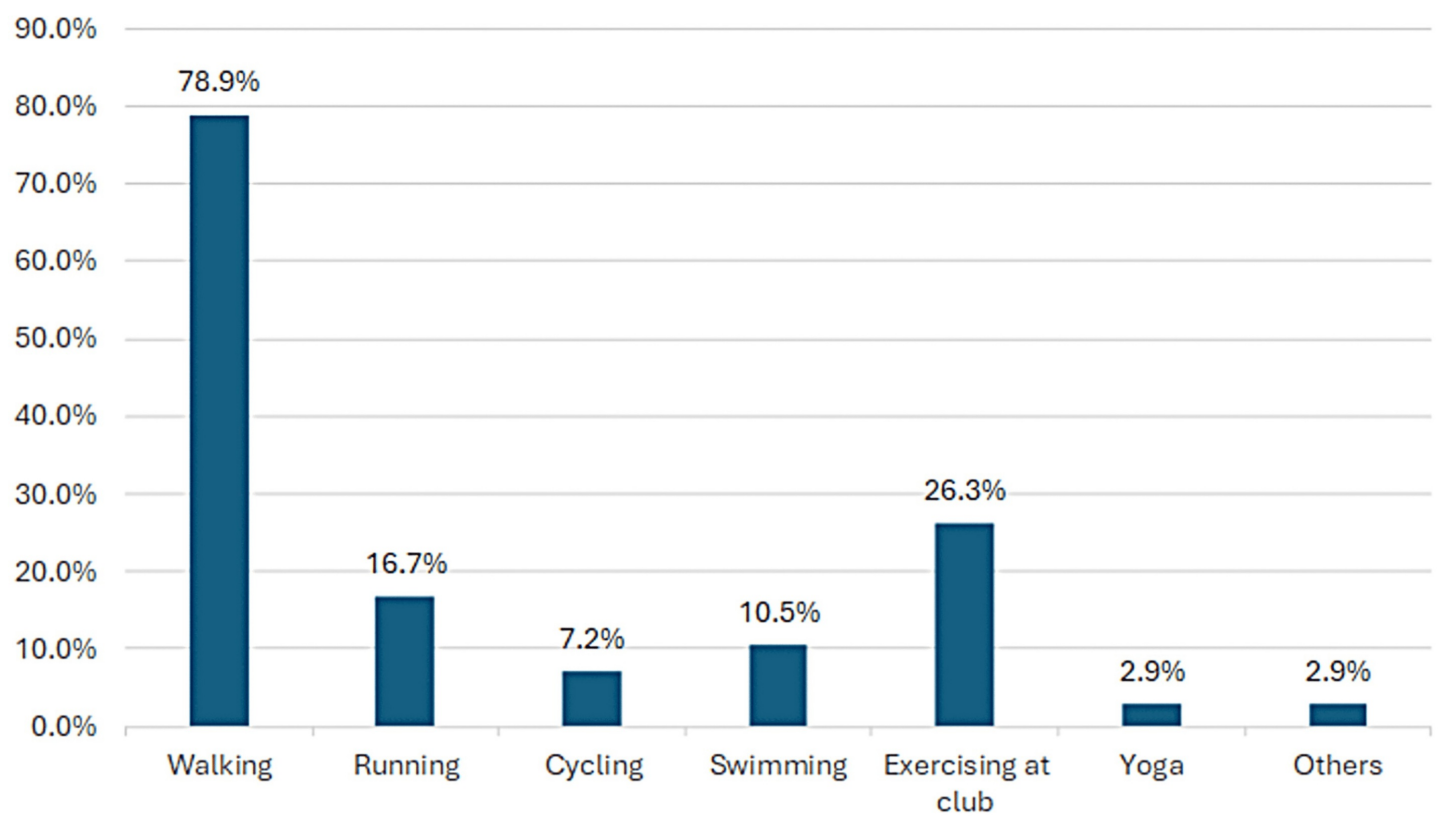
Types of physical activity practiced by the participants (n = 209)

Among the 381 participants, 209 (54.9%) reported practicing physical activity, while 172 (45.1%) did not. Among those who practiced physical activity, 165 (78.9%) engaged in walking, 55 (26.3%) exercised at a club, 35 (16.7%) practiced running, 22 (10.5%) participated in swimming, 15 (7.2%) did cycling, six (2.9%) practiced yoga, and six (2.9%) engaged in other activities (Figure 1).

**Figure 2:**
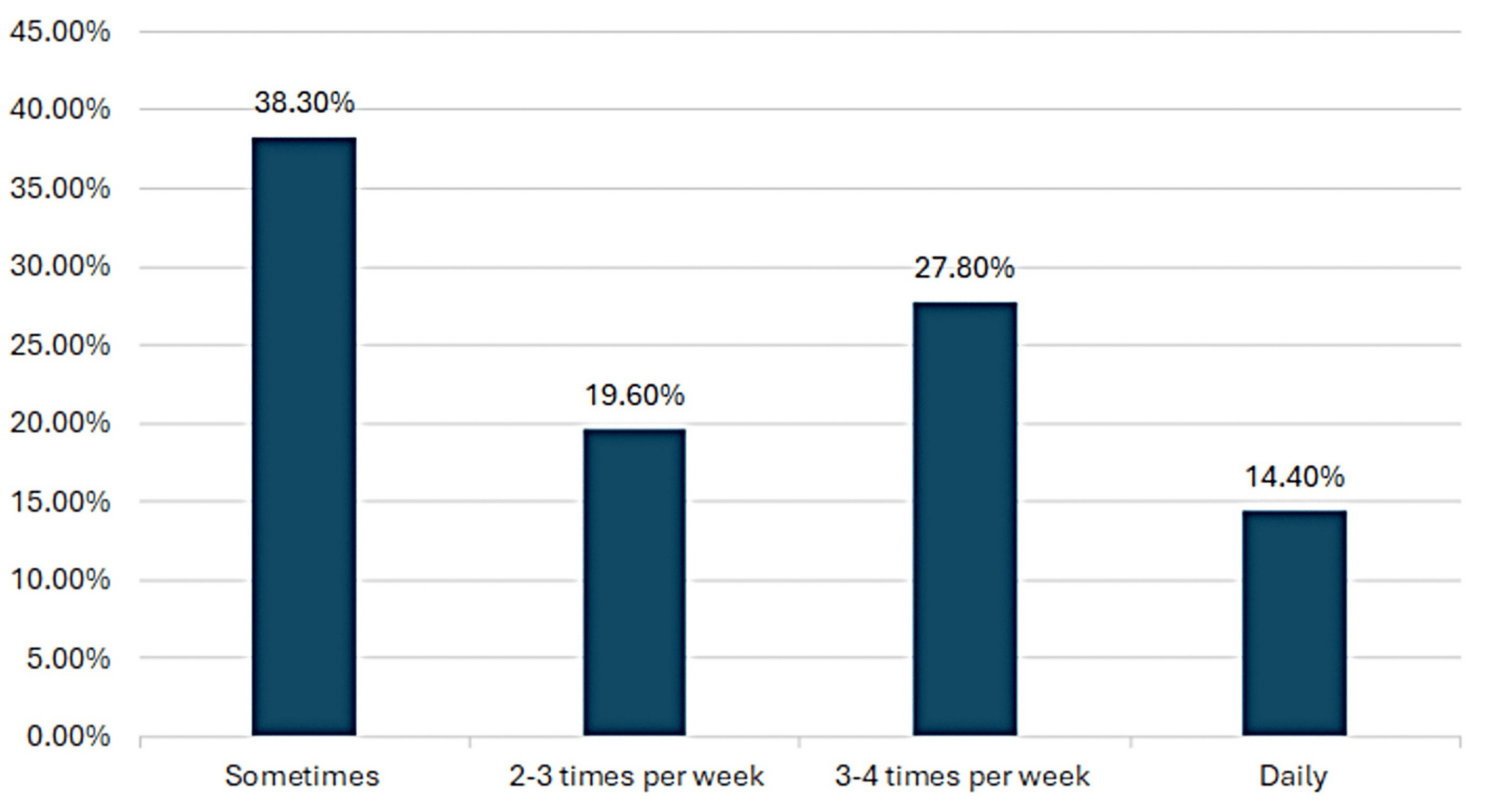
Frequency of practicing physical activity among the participants who reported being physically active (n = 209)

Eighty (38.3%) reported practicing physical activity sometimes, 41 (19.6%) practiced 2-3 times per week, 58 (27.8%) practiced 3-4 times per week, and 30 (14.4%) practiced daily, as shown in Figure 2.

The most commonly reported motivating factor was health benefits, cited by 184 (48.3%) participants, followed by weight management (166, 43.6%), blood sugar control (127, 33.3%), and personal pleasure (93, 24.4%). Physician's advice (74, 19.4%), social factors such as family or friends (39, 10.2%), and no answer (77, 20.2%) were less frequently reported as motivators. Regarding preventing factors, the most frequently cited barrier was lack of time (201, 52.8%), followed by lack of motivation (149, 39.1%) and physical limitations such as joint aches or fatigue (112, 29.4%). Some participants cited difficulty reaching exercise facilities (59, 15.5%) or other factors, such as old age or hot weather (8, 2.1%), while 56 (14.7%) gave no answer, as shown in Table 2. The associations between these factors and physical activity participation were analyzed using the Chi-square test, with p-values < 0.05 considered statistically significant.

**Table 2:** Factors that motivate or prevent patients to practice physical activity regularly (N = 381)

Factors	Frequency	Percentage
Motivating factors		
Health benefits	184	48.3
Weight management	166	43.6
Blood sugar control	127	33.3
Physician's advice	74	19.4
Social factors (family/friends)	39	10.2
Personal pleasure	93	24.4
No answer	77	20.2
Preventing factors		
Lack of time	201	52.8
Lack of motivation	149	39.1
Physical limitations (joint aches or fatigue)	112	29.4
Difficulty reaching utilities	59	15.5
Other factors (e.g., old age and hot weather)	8	2.1
No answer	56	14.7

About two-thirds of the participants, 250 out of 381 (65.6%), reported that they had carried out dietary changes to control diabetes, while 131 (34.4%) did not. The most frequent dietary change was lowering sugar and processed foods in the diet (187, 49.1%), followed by adopting a low-carbohydrate diet (145, 38.1%), avoiding certain foods such as fried and fast foods (111, 29.1%), increasing fiber intake (75, 19.7%), increasing fruit and vegetable intake (75, 19.7%), and eating more frequent small meals (67, 17.6%), as shown in Table 3. The associations between these dietary modifications and diabetes control practices were evaluated using the Chi-square test, with p-values < 0.05 considered statistically significant.

**Table 3:** Types of dietary changes carried out by the participants (n = 250)

Types of dietary change	Frequency	Percentage
Low-carbohydrate diet	145	38.1
More frequent small meals	67	17.6
More fiber in the diet	75	19.7
Low sugar and processed food diet	187	49.1
More fruit and vegetable intake	75	19.7
Avoiding some foods (e.g., fried and fast foods)	111	29.1

Table 4 presents the association between practicing sports and satisfaction with various aspects of life, including overall life satisfaction, perceived success, energy levels, and social life. A statistically significant association was found only for satisfaction with energy levels. Among those who did not practice sports, 60 (45.5%) reported dissatisfaction with their energy level compared to 30 (22.4%) participants who practiced sports, with an odds ratio of 2.89 (95% confidence interval (CI): 1.69-4.92, χ² = 13.97, p < 0.001), as determined by the Chi-square test. No significant associations were observed for satisfaction with life (χ² = 0.14, p = 0.712), satisfaction with success (χ² = 3.05, p = 0.080), or satisfaction with friends and social life (χ² = 0.96, p = 0.329), based on the corresponding odds ratios and Chi-square test results.

**Table 4:** Association between practicing sports and satisfaction with life, success, energy level, and health status *Statistically significant at p < 0.05 χ²: Chi-square value, df: degrees of freedom, OR: odds ratio, CI: confidence interval

Satisfaction domains	Practicing sports	Unsatisfied (number (%))	Satisfied (number (%))	OR (95% CI)	χ² (df)	p-value
Satisfaction with life	No	27 (20.5%)	105 (79.5%)	1.12 (0.61-2.06)	0.14 (1)	0.712
	Yes	25 (18.7%)	109 (81.3%)			
Satisfaction with success achieved	No	32 (24.2%)	100 (75.8%)	1.72 (0.93-3.18)	3.05 (1)	0.08
	Yes	21 (15.7%)	113 (84.3%)			
Satisfaction with energy level	No	60 (45.5%)	72 (54.5%)	2.89 (1.69-4.92)	13.97 (1)	<0.001*
	Yes	30 (22.4%)	104 (77.6%)			
Satisfaction with friends/social life	No	30 (22.7%)	102 (77.3%)	1.35 (0.74-2.46)	0.96 (1)	0.329
	Yes	24 (17.9%)	110 (82.1%)			

Adults aged 21-50 were significantly more likely to engage in physical activity (χ² = 17.15, p = 0.005), and activity was more common among those diagnosed with diabetes within the past 10 years (χ² = 10.92, p = 0.012). Active participants were also significantly more likely to report making dietary changes (χ² = 23.35, p < 0.001), engaging in stress control efforts (χ² = 10.37, p = 0.001), and achieving 6-8 hours of sleep per night (χ² = 16.38, p = 0.001). They more frequently believed in the effectiveness of exercise for diabetes control (χ² = 48.92, p < 0.001) and expressed greater confidence in managing diabetes through lifestyle modification (χ² = 20.22, p < 0.001). No statistically significant differences were found regarding gender, occupation, receiving healthcare advice, or perceived support from healthcare professionals (all p > 0.05), as shown in Table 5.

**Table 5:** Difference between participants who practice physical activity and those who do not practice physical activity *Statistically significant at p < 0.05 χ²: Chi-square value, df: degrees of freedom

Variables	Participants who do not practice physical activity (n = 172)	Participants who practice physical activity (n = 209)	χ² (df)	p-value
Age in years				
<20	14 (8.1%)	15 (7.2%)	χ² = 18.18 (5)	0.005*
21-30	18 (10.5%)	30 (14.4%)		
31-40	22 (12.8%)	50 (23.9%)		
41-50	41 (23.8%)	49 (23.4%)		
51-60	40 (23.3%)	45 (21.5%)		
>60	37 (21.5%)	20 (9.6%)		
Gender				
Male	97 (56.4%)	108 (51.7%)	χ² = 0.84 (1)	0.358
Female	75 (43.6%)	101 (48.3%)		
Occupation				
Student	23 (13.4%)	24 (11.5%)	χ² = 7.24 (4)	0.14
Employee (part time)	14 (8.1%)	28 (13.4%)		
Employee (full time)	38 (22.1%)	59 (28.2%)		
Employer	22 (12.8%)	29 (13.9%)		
Unemployed	75 (43.6%)	69 (33%)		
Duration of diabetes				
<1 year	34 (19.8%)	62 (29.7%)	χ² = 10.96 (3)	0.012*
1-5 years	35 (20.3%)	52 (24.9%)		
6-10 years	49 (28.5%)	56 (26.8%)		
>10 years	54 (31.4%)	39 (18.7%)		
Dietary changes				
No	82 (47.7%)	49 (23.4%)	χ² = 26.91 (1)	<0.001*
Yes	90 (52.3%)	160 (76.6%)		
Efforts to control stress				
No	117 (68%)	106 (50.7%)	χ² = 10.45 (1)	0.001*
Yes	55 (32%)	103 (49.3%)		
Average hours of sleep per night				
<4 hours	13 (7.6%)	12 (5.7%)	χ² = 15.89 (3)	0.001*
4-6 hours	31 (18%)	48 (23%)		
6-8 hours	70 (40.7%)	115 (55%)		
>8 hours	58 (33.7%)	34 (16.3%)		
Belief in exercise benefits				
No	2 (1.2%)	2 (1%)	χ² = 69.04 (2)	<0.001*
Not sure	65 (37.8%)	18 (8.6%)		
Yes	105 (61%)	189 (90.4%)		
Advice from healthcare provider				
No	35 (20.3%)	49 (23.4%)	χ² = 0.53 (1)	0.468
Yes	137 (79.7%)	160 (76.6%)		
Confidence in lifestyle modification				
Unconfident	12 (7%)	3 (1.4%)	χ² = 20.71 (3)	<0.001*
Neutral	29 (16.9%)	20 (9.6%)		
Confident	83 (48.3%)	90 (43.1%)		
Very confident	48 (27.9%)	96 (45.9%)		
Perception of support from healthcare professionals				
No	21 (12.2%)	28 (13.4%)	χ² = 2.00 (2)	0.368
Not sure	54 (31.4%)	60 (28.7%)		
Yes	97 (56.4%)	121 (57.9%)		

## Discussion

Our study demonstrates that most Saudi patients with type 2 diabetes in Al-Madinah are implementing lifestyle modifications, with over half reporting engagement in regular physical activity and nearly two-thirds making dietary changes to control their diabetes. These findings are consistent with several previous studies in the Kingdom, which show a growing awareness of the importance of lifestyle modification among diabetic patients [8,9]. In particular, walking was the most common form of exercise, aligning with national patterns and previous reports that walking is the preferred and most feasible activity in this population [10].

Our finding that 54.9% of the patients reported engaging in regular physical activity is higher than some previous studies, which reported adherence rates as low as 30%. This difference may be due to our study's broader inclusion criteria and a possible increase in patient awareness over time [9,10]. However, as in previous Saudi and international studies, a significant minority of patients remain inactive [8,11]. Major barriers identified by our participants, including lack of time, lack of motivation, and physical limitations, are echoed across multiple studies in the region [8,12].

Dietary change was also prominent among our cohort, with nearly half reducing sugar and processed food intake, and a substantial proportion lowering carbohydrate intake. This supports findings from previous Saudi and international research linking improved dietary habits with better diabetes outcomes [13,14]. However, studies continue to report a gap between knowledge and sustained behavioral change, especially among those with poor glycemic control [12,15].

A particularly important finding in our study was the positive association between physical activity and satisfaction with energy level, with active participants significantly more likely to feel satisfied with their energy. This supports physiological evidence linking exercise to improved metabolism and well-being, and agrees with similar associations found in other Saudi and Middle Eastern cohorts [11,14]. However, cross-sectional analysis cannot establish causality; it is possible that healthier, more energetic individuals are simply more likely to engage in exercise [8,16].

These results also highlight the clustering of healthy behaviors: patients who are physically active were more likely to also make dietary changes, manage stress, and report adequate sleep, findings supported by other local studies emphasizing the interconnected nature of diabetes self-management practices [8,13]. Interventions that address multiple lifestyle factors simultaneously may be more successful than those targeting individual behaviors in isolation.

Our findings echo calls in the Saudi literature for culturally sensitive educational and behavioral interventions. For example, Alzahrani et al. [8] and Choi et al. [12] both report that tailored education and community support can enhance self-management and glycemic control. However, as noted in multiple studies, structural barriers such as lack of access to facilities, limited family or social support, and persistent misconceptions continue to impede optimal lifestyle modification [8,9,12].

The strengths of this study include a relatively large sample size, focus on a culturally specific population, and integration of multiple domains of lifestyle modification.

However, limitations must be acknowledged. The cross-sectional design precludes causal inferences, the use of self-reported measures may introduce recall or social desirability bias, and the sample may not be representative of all Saudi diabetics, as the study was conducted at selected centers in Saudi Arabia, which may limit the generalizability of findings to other regions or populations. Furthermore, objective clinical outcomes (such as HbA1c) were not included, which limits the ability to directly link behavioral changes with glycemic control [8,13].

## Conclusions

This study highlights the growing adoption of lifestyle modifications among diabetic patients in Saudi Arabia, particularly in physical activity and dietary habits. While these trends are encouraging, significant barriers, such as a lack of motivation and environmental challenges, persist. Addressing these obstacles through culturally tailored, multi-level interventions is essential to support long-term diabetes management and improve patient outcomes.
